# Genetic Gains for Yield and Virus Disease Resistance of Cassava Varieties Developed Over the Last Eight Decades in Uganda

**DOI:** 10.3389/fpls.2021.651992

**Published:** 2021-06-21

**Authors:** Francis Manze, Patrick Rubaihayo, Alfred Ozimati, Paul Gibson, Williams Esuma, Anton Bua, Titus Alicai, Chris Omongo, Robert S. Kawuki

**Affiliations:** ^1^Department of Agricultural Production, Makerere University, Kampala, Uganda; ^2^National Crops Resources Research Institute (NaCRRI), Kampala, Uganda

**Keywords:** cassava breeding, cassava brown streak disease, cassava mosaic disease, yield related traits, genetic progress

## Abstract

Achieving food security for an ever-increasing human population requires faster development of improved varieties. To this end, assessment of genetic gain for key traits is important to inform breeding processes. Despite the improvements made to increase production and productivity of cassava in Uganda at research level, there has been limited effort to quantify associated genetic gains. Accordingly, a study was conducted in Uganda to assess whether or not genetic improvement was evident in selected cassava traits using cassava varieties that were released from 1940 to 2019. Thirty-two varieties developed during this period, were evaluated simultaneously in three major cassava production zones; central (Namulonge), eastern (Serere), and northern (Loro). Best linear unbiased predictors (BLUPs) of the genotypic value for each clone were obtained across environments and regressed on order of release year to estimate annual genetic gains. We observed that genetic trends were mostly quadratic. On average, cassava mosaic disease (CMD) resistance increased by 1.9% per year, while annual genetic improvements in harvest index (0.0%) and fresh root yield (−5 kg per ha or −0.03% per ha) were non-substantial. For cassava brown streak disease (CBSD) resistance breeding which was only initiated in 2003, average annual genetic gains for CBSD foliar and CBSD root necrosis resistances were 2.3% and 1.5%, respectively. It’s evident that cassava breeding has largely focused on protecting yield against diseases. This underpins the need for simultaneous improvement of cassava for disease resistance and high yield for the crop to meet its current and futuristic demands for food and industry.

## Introduction

Cassava (Manihot esculenta Crantz) is a major staple crop in the tropics (Food and Agriculture Organization of the United Nations (FAOSTAT), 2019) owing to its transformative potential to spur economic growth, rural development and food security (Otekunrin and Sawicka, 2019). Indeed, over 60% of the world’s cassava is produced in Africa (Food and Agriculture Organization of the United Nations (FAOSTAT), 2019), where its roots are processed into various forms (Shittu et al., 2016) to feed millions of people on a daily basis (Prakash, 2018). Within sub-Saharan Africa (SSA), cassava is recognized as a choice crop for climate change adaptation, as it performs reasonably well under prolonged droughts and marginal soils (Orek et al., 2020). It is for these reasons that cassava features predominantly in strategic plans for agricultural development of most SSA countries.

It suffices to note that cassava breeding efforts in Africa only began around 1930s (Storey and Nichols, 1938). During then, cassava mosaic disease (CMD) was a major breeding objective, as it had attained epidemic status on the continent (Legg and Thresh, 2000). Accordingly, pioneer cassava breeding efforts were initiated at Amani Research Station, Tanzania, to combat CMD. That breeding work involved interspecific hybridizations which led to the development and dissemination of cassava clones that were resistant to both CMD and cassava bacterial blight (Ortiz and Nassar, 2007).

The successful development of CMD resistant clones at Amani spurred an Africa-wide cassava research program that was instated at the International Institute of Tropical Agriculture (IITA) in Nigeria by 1971 (Hahn et al., 1980). Selected germplasm from Latin America, Asia and East Africa, along with cultivars from West Africa were collected to commence systematic genetic improvement of cassava at IITA (Hahn et al., 1980). Through that work, several elite genotypes with multiple resistances to prevalent pests and diseases and good culinary qualities were developed and disseminated to national breeding programs in Africa (Manyong et al., 2000).

In Uganda, CMD resistant varieties sourced from Tanzania formed a major part of the cassava production system between 1940s and 1980s (Otim-Nape et al., 2001), with clones such as Magana, Nyaraboke, Alado-Alado, Njure-Red, and Bamunanika predominating production in that period (Otim-Nape et al., 2001). It is should be noted that systematic cassava improvement in Uganda only started in the 1980s when a second wave of CMD caused by coinfection of African Cassava Mosaic Virus (ACMV) and the East African Cassava Mosaic Virus Uganda (EACMV-UG) emerged (Gibson et al., 1996; Patil and Fauquet, 2009). Subsequently, elite cassava clones combining yield and resistance to CMD were sourced from IITA and evaluated in Uganda to select those with durable CMD resistance. Through this process, some outstanding varieties including NASE 1, NASE 2, and NASE 3 were identified and promoted for production in the early 1990s (Ssemakula et al., 2000).

Released varieties were meant to be used for two main food products: “boiled or fried roots” that predominates central and western Uganda, and “flour-based meal” that predominates the eastern and northern parts of the country. As such, emphasis was initially placed on development of varieties characterized by high fresh root yield and dry matter content, multiple resistance to pests and diseases, starch quality, and low hydrogen cyanide (Ssemakula et al., 2000).

However, with the outbreak of cassava brown streak disease (CBSD) in early 2000s (Alicai et al., 2007), considerable efforts were diverted toward breeding for CBSD resistance, as the disease had then attained epidemic status and caused immense yield losses (Kawuki et al., 2016). CBSD damages the starch bearing part of cassava rendering it unfit for consumption, thereby causing huge economic losses and food insecurity (Hillocks et al., 2001). In fact, from the time when CBSD attained epidemic status in Uganda, cassava production in the country declined drastically from 4.9 million tons (MT) in the 2000s to the current 2.6 MT (Food and Agriculture Organization of the United Nations (FAOSTAT), 2019).

Another notable change in the 2010s, was the consideration of gender and integration of preferred end-user quality traits in cassava breeding operations (Esuma et al., 2019; Iragaba et al., 2019). Currently, cassava breeding in Uganda is designed to enhance key traits that contribute toward increased resilience, nutrition and productivity for the benefit of stakeholders involved in the production-processing-marketing-consumption continuum.

Through these breeding efforts, 21 cassava varieties have been released between 1993 and 2015, and several other elite clones developed using genomic selection (Ozimati et al., 2019). Despite the improvements made to increase production and productivity of cassava in Uganda at research level, there has been limited effort to quantify associated genetic gains. Quantifying such gains would guide cassava breeding processes, especially now when the rapidly increasing population demands faster development and deployment of improved varieties. Therefore, the objective of this study was to determine the rate of genetic gain per year for cassava traits that have been selected for between 1940 and 2019 in Uganda.

## Materials and Methods

### Plant Material

A total of 32 cassava varieties were used for this study (Table 1) and these were divided into four categories. Category one comprised local varieties; these arose from selections from Amani Research Program in Tanzania and were deployed for cultivation in Uganda between 1940s and1980s. Category two comprised varieties introduced from IITA and released in Uganda in the 1990s to combat CMD. Category three comprised a combination of varieties from IITA and Uganda; these were majorly developed for CMD resistance in the 2000s. Lastly, category four comprised varieties and elite clones developed in the 2010s to combat CBSD epidemic. All varieties were sourced from the Root Crops Research Program at the National Crops Resources Research Institute (NaCRRI) in Uganda, and had been maintained in Ngetta (northern Uganda), which is known to have low pressure of CBSD (Pariyo et al., 2015; Alicai et al., 2019). Sourcing planting materials from low disease pressure sites was important to ensure high vigor and uniform establishment.

**TABLE 1:** Summary of attributes and origin of varieties used for genetic gain assessment.

Code	Variety	Remarks	Status	Year	Special attributes at development and release
1	NASE 1	Introduced from IITA as TMS 60142	Released	1993	CMDt, high DMC, and low HCN
2	NASE 2	Introduced from IITA as TMS 30337	Released	1993	CMDt, good LR, and low HCN
3	NASE 3	Introduced from IITA as TMS 30572	Released	1993	CMDt, CBSDt, good LR, and low HCN
4	NASE 4	Introduction from IITA	Released	1999	CMDr and low HCN
5	NASE 5	Introduction from IITA	Released	1999	CMDt and low HCN
6	NASE 6	Introduced from IITA as TMS 4 (2) 1425	Released	1999	CMDr and low HCN
7	NASE 9	Introduced from IITA as 30555-17	Released	2003	CMDt, CBSDs, and low HCN
8	NASE 11	Introduced from IITA as 92/NA-2	Released	2003	CMDt, CBSDs, good LR, LUS, and low HCN
9	NASE 12	MH95/0414	Released	2003	CMDr, CBSDs, low HCN, and desirable CQ
10	NASE 13	MH97/2961	Released	2011	CMDr, CBSDs, high DMC, low HCN, and desirable CQ
11	NASE 14	MM96/4271	Released	2011	CMDr, CBSDt, high DMC, low HCN, and desirable CQ
12	NASE 15	Derivative of TME14	Released	2011	CMDr, CBSDt, high DMC, low HCN, and desirable CQ
13	NASE 16	Derivative of Bamunanika	Released	2011	CMDr, CBSDs, high DMC, low HCN, and desirable CQ
14	NASE 18	Derivative of TME14	Released	2011	CMDr, CBSDt, high DMC, low HCN, and desirable CQ
15	NASE 19	Derivative of TME14	Released	2011	CMDr, CBSDt, high DMC, low HCN, and desirable CQ
16	NAROCASS 1	NDL90/34HS	Released	2015	CMDr, CBSDt, high DMC, low HCN, and desirable CQ
17	NAROCASS 2	Introduced from Tanzania as MM06130	Released	2015	CMDr, CBSDt, high DMC, and desirable CQ
18	UG120124	MM96/4271//MH04/2767	Candidate	2019	CMDr, CBSDt, high DMC, and low HCN
19	UG110166	Introduction from Tanzania	Candidate	2019	CMDr, CBSDt, high DMC, and low HCN
20	UG120024	NASE 14/UG110043	Candidate	2019	CMDr, CBSDt, high DMC, and low HCN
21	UG120156	Introduction from Tanzania	Candidate	2019	CMDr, CBSDt, high DMC, low HCN, and high RWF
22	UG120183	Introduction from Tanzania	Candidate	2019	CMDr, CBSDt, high DMC, and low HCN
23	UG120198	Introduction from Tanzania	Candidate	2019	CMDr, CBSDt, high DMC, and low HCN
24	UG120193	Introduction from Tanzania	Candidate	2019	CMDr, CBSDt, high DMC, low HCN, and high RWF
25	UG110164	Introduction from Tanzania	Candidate	2019	CMDr, CBSDt, high DMC, and low HCN
26	Magana	Introduction from Tanzania	Landrace	1940	CMDt, quality flour and brew (popular in eastern Uganda)
27	Njure Red	Introduction from Tanzania	Landrace	1940	CMDt, soft when boiled or fried (popular in central Uganda)
28	Alado Alado	Introduction from Tanzania	Landrace	1940	CMDt, quality flour and brew (popular in northern Uganda)
29	Bamunanika	Introduction from Tanzania	Landrace	1940	CMDt, soft when boiled or fried (popular in central Uganda)
30	Bao	Introduction from Tanzania	Landrace	1940	CMDt, quality flour and brew (popular in northern Uganda)
31	Omo	Introduction from Tanzania	Landrace	1940	CMDt, EM, sweet, quality flour and brew (popular in west Nile)
32	Nyaraboke	Introduction from Tanzania	Landrace	1940	CMDt, soft when boiled or fried (popular in mid-western Uganda)

*IITA, International Institute of Tropical Agriculture; CMDt, tolerant to cassava mosaic disease; CMDr, resistant to cassava mosaic disease; CBSDt, tolerant to cassava brown streak disease; CBSDs, susceptible to cassava brown streak disease; DMC, dry matter content; RWF, resistant to whitefly; LR, leaf retention; LUS, long underground storage; CQ, culinary qualities; HCN, hydrogen cyanide; EM, early maturity. The candidate varieties (2019) have high CMD resistance, high DMC and high tolerance to CBSD.*

### Description of Trial Environments

All varieties were evaluated simultaneously at three environments representing major cassava agro-ecologies in Uganda, and this was done during the period April 2019 to May 2020. These environments were: Namulonge (0.5232°N, 32.6158°E), Serere (033°26′48.0″E, 01°32′22.6″N), and Loro (32°28′E, 2°12′N). Namulonge is located in the Lake Victoria crescent at an altitude of 1163 m above sea level (asl), and is characterized by reddish sandy-clay loam soils (Fungo et al., 2011). Serere is located in the semi-arid zones of eastern Uganda at an altitude of 1085 m asl with sandy loamy soils (Isabirye et al., 2004). Loro, on the other hand, has an altitude of 1063 m asl is also characterized by sandy loamy soils (Isabirye et al., 2004). Namulonge and Serere were specifically chosen because they are known to have high disease pressure for CMD and CBSD as well as high whitefly (vector) populations (Alicai et al., 2019). Loro was considered a suitable site for yield assessment owing to low disease pressure and vector populations for CBSD (Pariyo et al., 2015). The rainfall distribution at the three trial sites is bimodal with peaks in March to May and August to October, and mean annual precipitation ranges between 500 and 2800 mm, while temperature ranges between 15^0^C and 30^0^C (Nsubuga et al., 2014).

### Trial Design and Management

Trials at each site were planted in a randomized complete block design with two replications. Each clone was planted in five rows of six plants at 1 x 1 m spacing, making a plot size of 20 m^2^ with 30 plants. Adjacent plots were separated by 2-meter alleys to limit vegetative competition between varieties. Planting was done during the first growing season of 2019 (April) to ensure adequate soil moisture for sprouting. At 2 months after planting (MAP), six plants per plot were side-grafted with scions from highly infected TME 204, a standard CBSD susceptible check, to augment disease pressure for CBSD (Wagaba et al., 2013) at the three environments. All trials were conducted under standard agronomic practices for cassava (IITA, 1990).

### Data Collection

Data on disease incidence and severity for CMD and CBSD were collected on each plant in a plot at 3 and 6 MAP. CMD severity was assessed on a scale of 1–5; where 1 = no visible disease symptoms, 2 = mild chlorotic pattern on entire leaflets or mild distortion at base of leaflets, rest of leaflets appearing green and healthy, 3 = strong mosaic pattern on entire leaf, and narrowing and distortion of lower one-third of leaflets, 4 = severe mosaic, distortion of two-thirds of leaflets and general reduction of leaf size, and 5 = severe mosaic, distortion of four-fifths or more of leaflets, twisted and misshapen leaves (IITA, 1990). Similarly, CBSD foliar severity (CBSDfs) was scored on scale of 1–5, where 1 = no apparent symptoms, 2 = slight foliar chlorosis but with no stem lesions, 3 = pronounced foliar chlorosis and mild stem lesions with no die back, 4 = severe foliar chlorosis and severe stem lesions with no die back, and 5 = defoliation, severe stem lesions and die back (Gondwe et al., 2003).

At 12 MAP, trials were harvested to enable assessment of yield and other root attributes. All twelve plants within the net plot were harvested and partitioned into roots and above-ground biomass. Fresh root weight (FRW) and above-ground biomass were separately measured (kg plot^–1^) using a hanging weighing scale of 200 kg capacity. Harvest index (HI) was calculated as the ratio of FRW to total plant biomass as described by Kawano et al. (1978). Fresh root yield (FRY) (tones ha^–1^) was estimated by extrapolation of net plot root yields (Tumuhimbise et al., 2014). Root dry matter content (DMC) was determined by oven-drying of 100 g fresh samples at 80^o^C for 48 h, as described by Kawano et al. (1987). Lastly, data on cassava brown streak disease root necrosis incidence (CBSDri), and severity (CBSDrs) was recorded on all harvested roots/plot. Data on CBSDrs was collected using a standard scale of 1–5; where 1 = no observable necrosis, 2 = ≤ 5% of root necrotic, 3 = 6 to 25% of root necrotic, 4 = 26 to 50% of root necrotic with mild root constriction, and while 5 showed greater than 50% of root necrosis with severe root constriction (Gondwe et al., 2003).

### Data Analysis

For all measured traits, associated variance components were estimated by restricted maximum likelihood (Spilke et al., 2005). Effects of replicate nested in environment, variety, environment and variety by environment interaction were considered random, following the model below that was fitted using the lmer function in lme4 package (Bates et al., 2015) in R (R Core Team, 2019).

$$Y_{i\,j\,k}=\mu +\left(R_{j}\right)\,E_{k}+V_{i}+E_{k}+V\,x\,E_{i\,k}+e_{i\,j\,k}$$

where, *Y*_*ijk*_ = phenotypic value; μ overall mean; (*R*_*j*_)*E*_*k*_ = random effect of replicate *j* nested in *k^*th*^* environment such that *R_j_* ∼ *N*(0, σ^2^*_*j*_*); *V*_*i*_ = random effect of the *i*^*th*^ variety with *V_i_* ∼ *N*(0, σ^2^*_*i*_*); *E_k_* = random effect of *k*^*th*^ environment with *E_k_* ∼ *N*(0, σ^2^*_*k*_*); *VxE*_*ik*_ = random interaction effect of *i*^*th*^ variety with *k*^*th*^ environment such that *VxE*_*ik*_∼ *N*(0, σ^2^*_*ik*_*); and *e*_*ijk*_ random residual that is assumed to be normally distributed with mean zero and variance σ^2^. Respective broad sense heritability (*H*^2^)for each trait across environments was computed as:

$$H^{2}=\frac{\sigma_{V}^{2}}{\sigma_{v}^{2}+\frac{\sigma_{V\,x\,E}^{2}}{n}+\frac{\sigma_{e}^{2}}{r\,n}}$$

Where, $\sigma_{V}^{2}$ the variance component for variety; $\sigma_{V\,x\,E}^{2}$ = the variance for variety by environment interaction; $\sigma_{e}^{2}$ = the error variance; *n* = the number of environments; and *r* = the number of replications. Accordingly, best linear unbiased predictors (BLUPs) for each variety were extracted using the *ranef* function in *lme4* package (Bates et al., 2015). Eventually, BLUPs were used to perform correlation analyses, compute selection index and estimate annual genetic gains for evaluated traits, as they provide better estimates of genotype performance for unbalanced datasets than fixed clone effects (Piepho et al., 2008).

A weight-free rank summation index (RSI) (Hallauer et al., 1988; Badu-Apraku et al., 2013) was used to rank variety performances based on nine traits: FRY, HI, DMC, CMDs, cassava mosaic disease incidence (CMDi), CBSDfi, CBSDfs, CBSDri, and CBSDrs. To estimate genetic gains, BLUPs were assigned to the year when the variety was released i.e., varieties specifically released in 1940, 1993, 1999, 2003, 2011, 2015, and the current candidate varieties of 2019. Because released years were unevenly distributed, traits were regressed on order of release year i.e., 1, 2, 3, 4, 5, 6, and 7 representing 1940, 1993, 1999, 2003, 2011, 2015, and 2019, respectively. Effects of order of release year were tested for linear and quadratic responses of evaluated traits by orthogonal polynomial contrasts to determine the model that would best fit the set of data for a specific trait.

Absolute gain for linear relationships was obtained following the statistical model: *y* = *a*+*bx*,where; *y* is dependent variable; *x* = independent variable (order of released year); *a* = intercept; and *b* regression slope, which is the absolute genetic gain per released order (de Felipe et al., 2016). The slope was thereafter divided by the number of years for the respective breeding period to determine the annual genetic gain. Relative gain was obtained by dividing the absolute annual gain by mean trait performance of oldest released year that served as the check.

For quadratic relationships, absolute annual gain was calculated as the slope between two released orders i.e., between 1 (1940) and 2 (1993), 2 (1993) and 3 (1999), and 3 (1999) and 4 (2003), etc divided by the number of years for the respective breeding period. Relative gain was obtained by dividing the absolute annual gain by mean trait performance of older released year for each specific breeding period. Thus genetic gains were assessed sequentially in phases and as an average.

## Results

### Trait Heritabilities

Diseases (CMD and CBSD) and yield traits (DMC, HI, and FRYD) were differently affected by environment and genotypic effects (Table 2). For example, variety effects explained up to 96.4% of the total variance for CMD severity, while < 20% of total variance could be attributed to varieties for HI, DMC, and FRY. Indeed, highest heritability was registered for CMD (*H*^2^ = 0.96) and lowest registered for harvest index (*H*^2^ = 0.43). Overall, modest-high heritabilities i.e., H^2^ > 0.4 were observed for all evaluated traits (Table 2).

**TABLE 2:** Percentage of the total variance attributed to variety, environment and variety by environment interaction for evaluated traits.

Source of variation	CMDi	CMDs	CBSDfi	CBSDfs	CBSDri	CBSDrs	DMC	HI	FRY
Replicate/Environment	0.0	0.5	11.4	11.2	0.0	6.1	4.3	0.0	11.8
Variety	95.4	96.4	33.9	35.2	45.7	36.5	18.2	5.8	13.6
Environment	0.4	0.0	43.6	39.2	22.1	12.3	60.3	86.7	63.0
Variety*Environment	3.1	2.7	7.6	10.8	21.9	30.4	11.5	3.5	6.1
Residual	1.1	0.4	3.5	3.6	10.3	14.7	5.7	4.0	5.5
Genotype/Genotype*Environment	30.8	35.7	4.4	3.2	2.1	1.2	1.6	1.6	2.2
Broad-sense heritability (H^2^)	0.95	0.96	0.75	0.70	0.59	0.44	0.51	0.43	0.53

*CMDi, cassava mosaic disease incidence at 6 months after planting; CMDs, cassava mosaic disease severity at 6 months after planting; CBSDfi, cassava brown streak disease foliar incidence at 6 months after planting; CBSDfs, cassava brown streak disease foliar severity at 6 months after planting; CBSDri, cassava brown streak disease root incidence at 12 months after planting; CBSDrs, cassava brown streak disease root severity at 12 months after planting; DMC, root dry matter content; HI, Harvest index; FRY, Fresh root yield. Analysis based on data collected in 2019 at three sites; Namulonge (central region), Serere (eastern region) and Loro (northern region).*

### Performance of Varieties Based on Rank Summation Index

Based on RSI, the top performers were mostly candidate clones (UG120024, UG120193, UG120183, UG120198, and UG110164), and varieties officially released in 2011 (NASE 15), 1999 (NASE 4), 2015 (NAROCASS 1 and NAROCASS 2) and NASE 1 (1993) (Table 3). On the other hand, worst performers mostly comprised of popular local varieties (Magana, Nyaraboke, Bamunanika and Njure Red), and varieties released in 1993 (NASE 2 and NASE 3) or 2011 (NASE 13 and NASE 14). Both local varieties and varieties released in 1990s exhibited higher CMD and CBSD susceptibility compared to 2019 candidate clones or varieties released in 2015 (Tables 3, 4). Although varieties released in 2011 were generally resistant to CMD (incidence of ≤ 2.2%), they were susceptible to CBSD (severity ≥ 2 and incidence ≥ 41%). Candidate clones exhibited high tolerance/resistance to CMD and CBSD as well as high DMC (Tables 3, 4).

**TABLE 3:** Overall performance of clones based on rank summation index.

Genotype	Year of release	CMDi	CMDs	CBSDfs	CBSDfi	CBSDrs	CBSDri	DMC	HI	FRY	RSI	Rank
Alado Alado	1940	29	30	10	12	4	5	31	12	21	154	18
Bao	1940	28	31	6	6	20	17	30	20	18	176	22
Omo	1940	32	29	9	8	23	27	9	23	12	172	21
Bamunanika	1940	25	27	21	17	30	29	21	6	11	187	27
Njure Red	1940	30	32	29	28	10	11	13	8	20	181	23
Nyaraboke	1940	31	28	30	31	9	7	23	28	32	219	32
Magana	1940	27	24	23	26	17	19	11	28	30	205	30
NASE 1	1993	26	25	11	11	1	1	18	5	25	123	9
NASE 2	1993	21	22	26	22	21	26	24	13	8	183	24
NASE 3	1993	23	23	22	20	22	24	26	24	27	211	31
NASE 4	1999	12	9	15	19	4	6	28	2	5	100	6
NASE 5	1999	24	26	17	18	23	21	25	1	2	157	19
NASE 6	1999	13	15	31	27	16	20	27	9	26	184	26
NASE 9	2003	19	19	28	25	15	23	19	22	13	183	24
NASE 11	2003	20	20	14	14	25	22	10	4	1	130	11
NASE 12	2003	2	9	27	30	18	15	20	19	23	163	20
NASE 13	2011	10	11	32	32	32	32	5	30	10	194	29
NASE 14	2011	2	4	24	29	31	31	15	32	24	192	28
NASE 15	2011	2	5	13	13	19	16	6	20	4	98	4
NASE 16	2011	2	1	18	16	26	28	17	18	6	132	12
NASE 18	2011	1	3	25	24	29	30	7	15	14	148	17
NASE 19	2011	2	5	19	21	28	25	11	14	15	140	14
NAROCASS 1	2015	15	13	16	15	11	10	16	3	3	102	7
NAROCASS 2	2015	8	5	8	10	12	14	14	25	31	127	10
UG120193	2019	18	18	3	3	4	8	2	16	7	79	2
UG120024	2019	9	1	6	7	3	3	3	9	28	69	1
UG110164	2019	16	16	5	5	13	13	22	6	9	105	8
UG120183	2019	17	17	1	1	4	4	4	16	16	80	3
UG120124	2019	2	5	4	4	26	18	29	31	22	141	15
UG120156	2019	11	12	12	9	14	12	8	26	28	132	12
UG120198	2019	22	21	2	2	2	2	1	27	19	98	4
UG110166	2019	14	13	20	23	8	9	32	11	17	147	16

*CMDi, cassava mosaic disease incidence at 6 months after planting; CMDs, cassava mosaic disease severity at 6 months after planting; CBSDfi, cassava brown streak disease foliar incidence at 6 months after planting; CBSDfs, cassava brown streak disease foliar severity at 6 months after planting; CBSDri, cassava brown streak disease root incidence at 12 months after planting; CBSDrs, cassava brown streak disease root severity at 12 months after planting; DMC, root dry matter content; HI, Harvest index; FRY, Fresh root yield; RSI, rank summation index. BLUPs for disease traits (CMD and CBSD) based only on data from Namulonge and Serere owing to low disease pressure at Loro. Genotypes were ranked based on their BLUP values for each trait.*

**TABLE 4:** Means for cassava traits selected for between 1940 and 2019 in Uganda.

Year of release	No of Varieties	CMDs	DMC	HI	FRY	CBSDfs	CBSDrs
1940	7	3.5 ± 0.2	37.6 ± 1.1	0.37 ± 0.03	17.1 ± 3.5	1.8 ± 0.2	1.8 ± 0.3
1993	3	2.3 ± 0.3	37.4 ± 0.4	0.40 ± 0.003	20.9 ± 8.5	2.0 ± 0.3	1.8 ± 0.4
1999	3	1.6 ± 0.6	35.9 ± 0.6	0.45 ± 0.01	25.5 ± 7.4	2.1 ± 0.2	1.8 ± 0.3
2003	3	1.4 ± 0.2	38.5 ± 0.3	0.38 ± 0.01	25.6 ± 7.1	2.1 ± 0.2	2.1 ± 0.1
2011	6	1.1 ± 0.02	39.8 ± 0.6	0.30 ± 0.02	22.7 ± 2.5	2.2 ± 0.2	2.9 ± 0.2
2015	2	1.1 ± 0.1	38.8 ± 0.1	0.40 ± 0.07	17.5 ± 8.1	1.7 ± 0.5	1.5 ± 0.0
2019	8	1.3 ± 0.1	39.5 ± 1.3	0.30 ± 0.02	18.6 ± 1.8	1.3 ± 0.1	1.4 ± 0.2

*CMDs, cassava mosaic disease severity; DMC, dry matter content; HI, harvest index; FRY, fresh root yield; CBSDfs, cassava brown streak disease foliar severity; CBSDrs, cassava brown streak disease root severity.*

### Genetic Gains for Disease Resistance and Yield Related Traits

Cassava mosaic disease severity correlated negatively and significantly with order of release year (*r* = −0.9, *P* < 0.001) (Table 5). However, there was a negative non-significant correlation between CMD severity and CBSD foliar severity (*r* = −0.36, *P* = 0.13), and between CMD severity and CBSD root necrosis severity (*r* = −0.40, *P* = 0.08). CMD severity reduced from mean severity score of 3.5 (varieties released in1940) to 1.3 (candidate clones of 2019), attaining an average annual genetic gain of 1.9% (Table 6). Highest annual gains were registered for 1993 to 1999 (5.2%) and 1999 to 2003 (8.1%). However, CMD susceptibility increased by 4.5% per year from 2015 to 2019.

**TABLE 5:** Pearson’s correlation coefficients among selected cassava traits evaluated in cassava varieties released between 1940 and 2019 in Uganda.

	DMC	CMDs	CBSDfs	CBSDrs	HI	FRY
CMD6s	–0.21	1				
CBSDfs	–0.29	–0.36	1			
CBSDrs	–0.17	–0.40	0.71***	1		
HI	–0.21	0.07	0.03	–0.34	1	
FRY	0.06	–0.08	0.17	0.22	0.58***	1
Order of release year	0.40*	−0.9***	−0.74***	−0.63**	–0.11	0.07

CMD6s, cassava mosaic disease severity at 6 months after planting; CBSDfs, cassava brown streak disease severity at 6 months after planting; CBSDrs, cassava brown streak disease root necrosis severity at 12 months after planting; DMC, dry matter content; HI, harvest index; FRY, fresh root yield; *P < 0.05, **P < 0.01; ***P < 0.001. Correlations including CBSDfs and CBSDrs were performed using varieties or clones developed between 2003 and 2019 because selection for CBSD resistance began in 2003. Two sets of correlations were performed: (1) between evaluated traits and order of release year, and (2) amongst the evaluated traits across the released years.

**TABLE 6:** Genetic gains for cassava traits selected for between 1940 and 2019 in Uganda.

Breeding period			Absolute annual gain					Relative annual gain (%)				
		
From	To	No. of years	CMDs	DMC	CBSDfs	CBSDrs	FRY	CMD	DMC	CBSDfs	CBSDrs	FRY
1940	1993	53	−0.02	0.004	0.004	0.004	2.6 kg	−0.57	0.01	0.22	0.22	0.02
1993	1999	6	−0.12	0.033	0.010	0.010	20 kg	−5.20	0.09	0.50	0.56	0.10
1999	2003	4	−0.13	0.050	0.000	0.000	15 kg	−8.10	0.14	0.00	0.00	0.06
2003	2011	8	−0.03	0.025	−0.010	−0.008	0.0 kg	−1.80	0.06	−0.48	−0.38	0.00
2011	2015	4	0.00	0.050	−0.050	−0.033	−30 kg	0.00	0.13	−2.30	−1.10	−0.13
2015	2019	4	0.05	0.050	−0.070	−0.047	−38 kg	4.50	0.14	−4.10	−3.10	−0.21
Average genetic gain			−0.04	0.035	−0.04	−0.03	−5 kg	−1.90	0.10	−2.30	−1.50	−0.03
Adjusted *R*^2^ linear			0.63	0.16	0.14	0.02	0.02					
Adjusted *R*^2^ quadratic			0.85	0.10	0.41	0.18	0.079					

*CMDs, cassava mosaic disease severity at 6 months; DMC, dry matter content; CBSDfs, cassava brown streak disease foliar severity at 6 months after planting; CBSDrs, cassava brown streak disease root severity at 12 months after planting; FRY, fresh root yield; *R*^2^, coefficient of determination of the relationship between order of release year and the changes in traits over the years. Average annual gains (absolute and relative) for resistance to CBSD were computed using estimates from 2003 to 2019 because selection for the trait only began in 2003. There were no genetic gains for harvest index between 1940 and 2019. With the exception of DMC where genetic gains were estimated using slope of linear regression, annual genetic gains for all other traits were estimated using the slope between two released orders from the quadratic graphs, because the quadratic model provided higher *R*^2^ values.*

For CBSD, we observed negative significant correlations between order of release year and CBSD foliar severity (*r* = −0.74, *P* < 0.001). Similar observations were made between order of release year and CBSD root necrosis severity (*r* = −0.63, *P* < 0.01) (Table 5). CBSD foliar severity correlated positively and significantly with CBSD root necrosis severity (*r* = 0.67, *P* < 0.01). CBSD foliar severity reduced from symptom severity score of 2.1 in 2003 to 1.3 in 2019 and thus attaining an average annual genetic gain of 2.3% (Table 6). Highest annual gains were recorded for 2015 to 20.19 (4.1% per year). Similarly, CBSD root necrosis severity reduced from root necrosis score of 2.1 in 2003 to root necrosis score of 1.4 in 2019 and thus attaining an average annual genetic gain of 1.5%.

**FIGURE 1:**
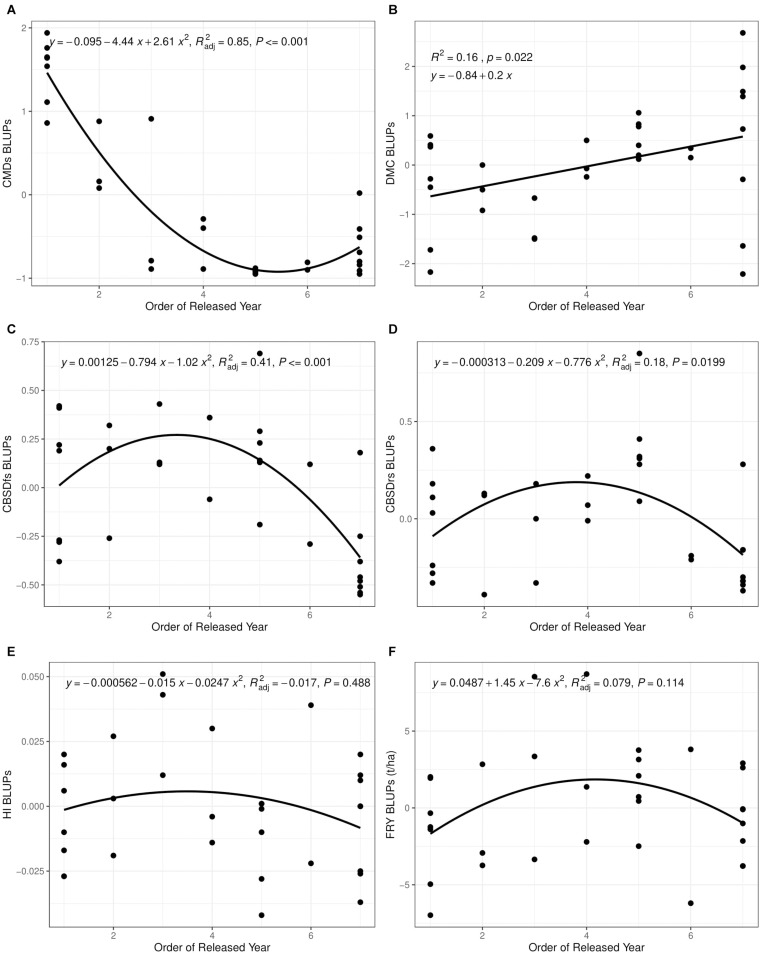
Changes in yield and disease severity for 32 varieties released and/or developed between 1940 and 2019 in Uganda. **(A)** Cassava mosaic disease severity at 6 months after planting (CMDs). **(B)** Dry matter content (DMC). **(C)** Cassava brown streak disease foliar severity at 6 months after planting (CBSDfs). **(D)** Cassava brown streak disease root necrosis severity at 12 months after planting (CBSDrs). **(E)** Harvest index (HI). **(F)** Fresh root yield (FRY). CBSD resistance breeding was initiated in fourth released year (2003).

Much as order of release year correlated positively and significantly with dry matter content (*r* = 0.40, *P* = 0.02), we observed small, positive, nonsignificant correlations between order of release year and harvest index (*r* = −0.11, *P* = 0.53), plus fresh root yield (*r* = 0.07, *P* = 0.73). Fresh root yield correlated positively and significantly with harvest index (*r* = 0.58, *P* < 0.001). From 1940 to 2019, root dry matter content increased linearly from 37.6 to 39.4% with a genetic gain of 0.1% per year. Fresh root yield increased from 17.1 tons per ha in 1940 to 25.5 tons per ha in 1999 with an average annual gain of 0.06%. However, fresh root yield reduced from 25.6 tons/ha in 2003 to 18.6 tons/ha in 2019 at a rate of 0.12% per year (Table 6). Meanwhile, there were no genetic gains for harvest index between 1940 and 2019 (Figure 1).

## Discussion

Development and deployment of nutritious, stress-resilient, and high yielding cassava varieties requires identification and introgression of desirable alleles. As part of this process, routine assessment of genetic gain for key traits is necessary to identify gaps and quantify progress made toward attainment of prior defined breeding targets. Among the various methods for genetic gain assessment, growing released varieties in a common set of environments and regressing their trait means on year of release has gained popularity, as germplasm from recurrent selection programs is rarely available in breeding programs (Rutkoski, 2019). Accordingly, in this study, cassava varieties developed in Uganda between 1940 and 2019, were evaluated in 2019 to get insights into annual genetic gains. This was the first attempt to estimate genetic gain for selected cassava traits in Uganda.

Significant genotype variances were observed for all evaluated traits and thus, positively confirming the appreciable genetic variability in the evaluated clones and varieties (Ssemakula et al., 2000). The high heritabilities observed for disease traits are comparable to heritability estimates by Okul et al. (2018) and suggest that Namulonge (central Uganda) and Serere (eastern Uganda) are areas of high disease pressure for CMD and CBSD. Consistency in variety or clone rankings for CMD resistance could imply durability of resistance in the tested genotypes. Indeed, local and varieties released in 1990s consistently registered higher CMD susceptibility when compared to recent elite clones or varieties released in 2015 (Table 3). However, variety or clone rankings for CBSD resistance were not consistent across environments, possibly because there could be different cassava brown streak virus strains that are resident in test environments. Cassava brown streak viruses [cassava brown streak virus (CBSV) and Uganda cassava brown streak virus (UCBSV)] have been reported to evolve rapidly, a phenomenon that could influence virulence (Ndunguru et al., 2015; Alicai et al., 2016) and thus amplify genotype by environment interactions (Pariyo et al., 2015; Okul et al., 2018). These findings further underpin the need for systematic evaluation and screening for CBSD in locations that are truly hotpots so as to discern resistant from susceptible clones.

Generally, candidate varieties and recently released varieties exhibited higher disease resistance (Tables 3, 4). Indeed, some of the candidate varieties e.g., UG120156 and UG120024 have also been reported by Okul et al. (2018) to exhibit high CBSD resistance. One possible explanation for this is that these candidate clones and/or varieties were selected for dual resistances to CMD and CBSD, which was not the case with varieties released before 2011. An exceptional clone was NASE 4, a variety released in 1999, which ranked among the top 10 performers; its ability to maintain superior and stable performance over a wide range of environments could explain this trend (Adriko et al., 2011).

Local varieties such as Magana (popular in eastern region), Nyaraboke (popular in mid-western region), Bamunanika (popular in central region) and Njure Red (popular in central region), were among the worst performers. These varieties showed high susceptibility to both CMD and CBSD (Tables 3, 4). It is important to note that these local varieties were among the first CMD resistant clones developed in 1930s in Amani (Tanzania) and introduced into Uganda in the 1940s for cultivation (Legg and Thresh, 2000). These clones were deployed for production in 1950s and formed a major part of the cassava production system in Uganda until the 1980s (Otim-Nape et al., 2001), when a second wave of CMD caused by co-infection of African Cassava Mosaic Virus (ACMV) and the recombinant strain of the East African Cassava Mosaic Virus (EACMV-UG) emerged (Patil and Fauquet, 2009). The breakdown of CMD resistance in local varieties and varieties released in early 1990s (Table 3) is likely due to the long exposure to viruses or synergistic infections from the different cassava mosaic germiniviruses (CMGs).

Following the CBSD outbreak in Uganda in the early 2000s (Alicai et al., 2007), efforts were initiated to develop and release varieties that combine both CMD and CBSD resistance. The first batch of these varieties were officially released in 2011, all in an effort to limit spread and damage inflicted by CBSD. Notable of these were: NASE 14, NASE 15, NASE 16, NASE 18, and NASE 19. However, in the present study, these varieties maintained CMD resistance, but succumbed to CBSD, as exhibited in their respective CBSD foliar incidence (Table 4). Given that this assessment was done 8 years after these varieties were released, it is likely that the high root necrosis severity scores (Tables 3, 4) are a reflection of increased virus load accumulating in the vegetative tissues during this propagation period (Shirima et al., 2019). Similar observations were made by Mukiibi et al. (2018) and Okul et al. (2018), who reported that NASE 14 (released in 2011) registered high CBSD foliar and root incidence and severity after 6 years of release. This situation may be attributed to changes in the composition of virus species and/or virulence that overwhelms host defense systems and cause resistance breakdown or degeneration (Shirima et al., 2019). The clonal nature of cassava propagation amplifies this problem.

Correlation analyses were performed to assess relationships between order of release year and traits evaluated (Table 5). The significant linear relationships between order of release year and CMD resistance plus dry matter content suggest that breeding efforts between 1940 and 2019 were successful in developing CMD resistant genotypes with high dry matter content. Significant negative correlations between order of release year and CBSD resistance also suggest that breeding efforts undertaken between 2003 and 2019 majorly focused on development and/or release of CBSD resistant varieties. The small nonsignificant correlations between order of release year and yield-related traits (FRY and HI) are indicative of preferential selection and release of genotypes with more emphasis placed on disease resistances as compared yield.

Direct selection for disease resistance without similar efforts devoted to yield-traits could explain the non-significant positive correlations between CBSD resistance with FRY or HI. On the other hand, high significant positive correlation between CBSD foliar and CBSD root necrosis severity, could imply that both traits were directly selected for, as witnessed by their respective reductions across years of release. Negative nonsignificant correlations between CBSD resistance and CMD resistance between 2003 and 2019, could suggest that high levels of CMD resistance had been attained at the time when selection for CBSD resistance was initiated, and therefore, most of the clones were tolerant to CMD, but had not attained similar levels of resistance for CBSD.

Based on regression analyses, CMD severity reduced by an average of 1.9% per year between the period 1940 and 2019. This genetic gain estimate is higher than that provided by Okechukwu and Dixon (2008), who reported 0.65% genetic gain per year for CMD resistance among IITA clones developed in Nigeria between 1970 and 2000. The highly significant genetic gain per year for CMD resistance could be explained in three ways. Firstly, breeding efforts targeting CMD resistance have been ongoing since 1930s (Legg and Thresh, 2000), which is sufficient time for increasing the frequency of resistance alleles in the breeding population through recurrent selection (Hallauer et al., 1988). Secondly, CMD resistance is largely governed by additive genetic effects (Hahn et al., 1980; Wolfe et al., 2016; Rabbi et al., 2020), which makes it amenable to genetic gains from recurrent selection. Thirdly, that deployed CMD resistance was effective against the prevalent cassava mosaic germiniviruses. Indeed, latest findings by Mukiibi et al. (2018) have showed that both single and coinfection of ACMV and EACMV-UG do exist in Uganda. The 4.5% increase in CMD susceptibility between 2015 and 2019 may be attributed to tradeoffs during selection for combined resistance to CBSD and CMD or use of CBSD resistant parents that are deficient in CMD resistance.

Much as research efforts to combat CBSD began in early 2000s when the disease had attained epidemic status in Uganda (Alicai et al., 2007), some varieties like NASE 1 that were released in 1993, exhibited high CBSD tolerance (Table 3). This finding could indicate that CBSD resistance alleles were present in IITA germplasm, from which NASE 1 was derived. Since 2003 when systematic CBSD resistance improvement began, there were average genetic gains of 2.3% per year for CBSD foliar resistance, and 1.5% per year for CBSD root necrosis resistance (Table 6). These genetic gains for CBSD resistance within such a relatively short timeframe could be attributed to the concerted and systematic approaches taken to harness and utilize available genetic resources in cassava breeding (Abaca et al., 2012; Kaweesi et al., 2014; Pariyo et al., 2015; Kawuki et al., 2016; Okul et al., 2018; Ozimati et al., 2018). Predominance of additive gene effects for CBSD resistance (Kulembeka et al., 2012; Chipeta et al., 2018), which can be exploited through recurrent selection, have equally enabled consolidation of gains.

Between 1940 and 2019, generally 5 kg per ha per year were lost for fresh root yield and no genetic gains in harvest index were observed; equally low genetic gains were recorded for dry matter content (0.1% per year) (Table 6). This is contrary to findings from earlier studies by Okechukwu and Dixon (2008), and Ceballos et al. (2020), who reported annual genetic gains of 1.2% and 1.0% for fresh root yield in Nigeria and Thailand, respectively. Differences in selection strategies customized to address local needs in Uganda, Nigeria and Thailand could explain this variation. For example, breeding programs in South East Asia have for long, mainly focused on developing cassava clones with high yield and root quality traits such as starch (Ceballos et al., 2020). Similarly, cassava breeding programs in West Africa (Nigeria) have focused on development of cassava clones that combine high fresh root yield, root quality and CMD resistance (Manyong et al., 2000). In Uganda, however, critical traits selected for include; dual resistance to CMD and CBSD, high yield and desirable root quality (Kawuki et al., 2016). Certainly, selection for several traits limits genetic progress as it leads to compromising tradeoffs amongst target traits. For example, before CBSD emerged in Uganda, fresh root yield increased from 17.1 tons in 1940 to 25.6 tons in 2003 (Table 4). However, when CBSD attained epidemic status in the early 2000s, fresh root yield reduced from 25.6 tons in 2003 to 18.6 tons in 2019. Another good example is the sharp contrast between fresh root yield and CBSD resistance observed in clones UG120024 and UG120156 (Table 3).

## Conclusion

The study described herein was conducted to estimate annual genetic gains for critical cassava traits that have been selected for between 1940 and 2019 in Uganda. Based on the generated datasets, this study revealed that there was significant annual genetic improvement of cassava for resistance to CMD and CBSD. Findings from the present study also demonstrated that the annual rate of genetic gain for cassava yield in Uganda is not sufficient to achieve the desired output necessary to reach the cassava production demand predicted for 2050. This underpins the urgent need to incorporate simultaneous selection for disease resistance and high yield for the crop to meet its current and futuristic demands for food and industry.

## Data Availability Statement

The original contributions presented in the study are included in the article/Supplementary Material, further inquiries can be directed to the corresponding author.

## Author Contributions

FM involved in data collection, analysis, and the manuscript writing. PR and PG involved in review and supervision. AO and WE involved in data collection, the manuscript editing, and review. AB and CO involved in review. TA involved in the manuscript review and editing. RK involved in data collection, analysis, the manuscript editing, and review and funding acquisition. All authors contributed to the article and approved the submitted version.

## Conflict of Interest

The authors declare that the research was conducted in the absence of any commercial or financial relationships that could be construed as a potential conflict of interest.
